# Production of Lightweight Alkali Activated Mortars Using Mineral Wools

**DOI:** 10.3390/ma12101695

**Published:** 2019-05-24

**Authors:** Ahmad Alzaza, Mohammad Mastali, Paivo Kinnunen, Lidija Korat, Zahra Abdollahnejad, Vilma Ducman, Mirja Illikainen

**Affiliations:** 1 Fibre and Particle Engineering, Faculty of Technology, University of Oulu, 90014 Oulu, Finland; Ahmad.Alzaza@oulu.fi (A.A.); Paivo.Kinnunen@oulu.fi (P.K.); Zahra.abdollahnejad@oulu.fi (Z.A.); Mirja.Illikainen@oulu.fi (M.I.); 2 National Building and Civil Engineering Institute, Dimičeva ulica 12, SI-1000 Ljubljana, Slovenia; lidija.korat@zag.si (L.K.); vilma.ducman@zag.si (V.D.)

**Keywords:** lightweight alkali activated mortar, mineral wool, fresh properties, mechanical properties, durability properties, drying shrinkage

## Abstract

This experimental study aimed to develop a fiber-reinforced lightweight mineral wool-based alkali activated mortar. The lightweight mineral wool-based alkali activated mortars were produced using premade foam and reinforced by polypropylene (PP) fibers. They were assessed in terms of fresh and hardened-state properties. Fresh-state properties were investigated by mini-slump tests. Hardened-state characteristics were assessed by ultrasonic pulse velocity, dry density, compressive and flexural strengths, drying shrinkage, efflorescence, water absorption, and permeable porosity. For the first time, the resistance of the synthesized lightweight mineral wool-based alkali activated mortars against harsh conditions (carbonation, freeze and thaw, and high temperature) were evaluated. The porous structures of the developed lightweight alkali activated mortars were also analyzed using an X-ray micro-computed tomography (CT) technique. Lightweight mix compositions with densities in a range of 770–1510 kg/m^3^, compressive strengths of 1–9 MPa, and flexural strengths of 2.6–8 MPa were developed. Increases in both density and strength after carbonation were also recorded, while a decrease of strength was noticed after exposure to freeze/thaw and high temperatures of up to 500 °C.

## 1. Introduction

Increasing environmental awareness has resulted in pressure on the concrete industry to decrease its CO_2_ emissions and resource consumption. One approach to decreasing the environmental impact of the production of concrete is to utilize industrial side-streams instead of cement [1,2,3,4,5]. Alkali-activated materials (AAM) are a potential replacement for the ordinary Portland cement (OPC) based binders, with an indicated environmentally friendly process and acceptable mechanical and durability properties [1,2,3,4,5,6]. A wide range of aluminosilicate sources has been investigated as precursors in AAM, including widely studied fly ash (FA), ground granulated blast-furnace slag (GGBFS), and metakaolin (MK) [7,8]. One of the materials getting increasing attention for use in the production of alkali-activated cementitious binders is mineral wool [9]. Mineral wools (an umbrella-term for glass wool and stone wools) are widely used as insulation materials in buildings, and they usually end up in landfills after the buildings have been torn down [9]. The research related to alkali-activated mineral wools is in its initial stage, even if promising results (compressive strength of 30–70 MPa) of alkali-activated mineral wool-based binders have been achieved [9,10].

Lightweight AAM can combine the benefits of alkali activation technology with lightweight cementitious compositions. It provides construction materials with lower environmental load in terms of resources consumption and CO_2_ emissions [11]. Moreover, due to the ability of lightweight cementitious compositions to decrease the operational energy consumption in buildings owing to its insulation properties, it has received increasing interest [12].

Introducing air voids to alkali-activated binders can be implemented either mechanically (using premade foam) or chemically (using foaming agents such as either aluminum (Al) or hydrogen peroxide (H_2_O_2_)) [1,13,14,15,16]. The mechanical foaming method was favored over the chemical method due to the following advantages: (1) using a lower amount of foaming agent with a lower environmental footprint; (2) higher degree of pore circularity; (3) smaller pore size and narrower pore size distribution; (4) lower tendency for air voids to collapse or merge [14,17]. However, chemically fabricated lightweight AAM exhibited a lower density compared to those synthesized mechanically [18].

Generally, lightweight AAM achieved a low density (150–2200 kg/m^3^) with a compressive strength ranging in an average of 0.2–30 MPa. However, lightweight OPC-based compositions showed density and compressive strength in the ranges of 360–1400 kg/m^3^ and 1–10 MPa, respectively [1,19]. The strength of lightweight OPC-based compositions decreases with a reduction in density [1]. However, no correlation was detected between compressive strength and density in lightweight FA-based AAM [15]. Additionally, it was demonstrated that higher pore circularity, smaller pore size, and narrower pore size distribution resulted in a higher compressive strength in lightweight AAM [14,18,19,20,21].

Moreover, it was detected in [22,23,24] that curing the lightweight AAM under 60 °C for 24 h, followed by open-air curing, increased the compressive strength and reduced porosity and water absorption as compared to the samples cured only in open-air conditions. However, at curing temperatures higher than 60 °C, a reduction in compressive strength of the lightweight MK/FA-based AAM was detected [24]. In contrast, the compressive strength of the lightweight FA-based AAM cured at 85 °C was higher than those cured at 55 °C [25]. Curing at elevated temperatures increased the geopolymerization rate: the lightweight AAM that was cured at 60 °C for 24 h obtained approximately 97% of its 28-day strength after seven days, while the samples cured at room temperature gained only 75% [23,26,27].

Several studies have investigated the resistance of lightweight AAM under different harsh conditions, such as high temperatures [22,28,29,30,31,32]. Lightweight AAM demonstrates a special behavior in terms of compressive strength after exposure to high temperatures (100–1200 °C) as compared to normal-weight AAM (without foam). Interestingly, Zhang, et al. [22] indicated that lightweight FA-based AAM gained strength after exposure to temperatures of up to 800 °C, where a 100% increase in strength was recorded. The strength retention observed after heating to 800 °C was attributed to the high-temperature stability of Al-Si rich gels of low-calcium in FA-based AAM. In contrast, a decrease in the compressive strength of the lightweight FA-based AAM was measured by exposing it to high temperatures of up to 800 °C [29]. However, in the same study [29], an increase in the compressive strength of the lightweight FA-based AAM was detected after heating to 1000 °C because of its sintering.

Limited experimental results were found related to lightweight concrete resistance against accelerated carbonation [31,32] and freeze-and-thaw cycles [33]. Amran et al. [13] indicated that foamed concrete exhibited high resistance to accelerated carbonation. Jones et al. [31] reported a directly proportional relationship between carbonation resistance and the density of lightweight OPC/FA concrete. Furthermore, the porosity of the lightweight concrete can affect freeze/thaw resistance, depending on the open permeable porosity. For instance, the closed pore structure leads to lower permeable porosity and water absorption and therefore lower damage due to the increased volume of frozen water at freeze/thaw conditions. Positive effects of the closed pore structure on increasing freeze and thaw resistance were detected in [33].

In practice, mineral wools as construction waste and demolished materials are commonly found mixed during the demolishing and disposal, because the cost of separation process could be avoided. Minerals wools have principal differences in their chemical compositions, where calcium oxide (CaO) and alumina (Al_2_O_3_) contents are higher in stone wool, while glass wool contains a higher silica (SiO_2_) content. Therefore, the use of mineral wools brings the economic and environmental benefits, however, a knowledge gap was detected in the utilization of the minerals wools in alkali activation technology foamed concrete to develop lightweight indoor panels. Therefore, to fill this knowledge gap, this experimental study aimed to develop fiber-reinforced lightweight mineral wool-based alkali activated mortar using different premade foam contents. In all compositions, metakaolin was added as a source of silicon and aluminum to mineral wools in order to promote durable end-product formations. The lightweight mineral wool-based alkali activated mortar was reinforced with polypropylene (PP) fibers to enhance the mechanical properties of AAM [11,34,35,36]. Thereafter, the developed fiber-reinforced lightweight mineral wool-based alkali activated mortar was assessed in terms of its density, ultrasonic pulse velocity (UPV), mechanical properties, drying shrinkage, efflorescence, water absorption, and permeable porosity. Furthermore, resistance against harsh conditions such as carbonation, freeze and thaw, and high temperatures were investigated. In addition, the impacts of the harsh conditions on the porous structure characteristics were compared using X-ray micro-computed tomography (CT) scanning technique.

## 2. Experiment Plan

### 2.1. Materials and Mix Design

Fiber-reinforced lightweight alkali activated mortar was comprised of stone wool (SW), glass wool (GW), metakaolin (MK), sand, alkali activator (a solution of sodium silicate and sodium hydroxide), PP fibers, and premade foam. Stone wool was supplied by Paroc Group Oy, Helsinki, Finland, in a fibrous form. The fibrous raw material has been milled into a powder with a particle size distribution (d_50_ ≤ 11 µm). The milling was done in a 10 L tumbling ball mill filled with 137 metallic balls with a diameter of 25 mm each. One milling cycle consists of five portions (200 g/portion) and lasts for 100 min. GW (d_50_ ≤ 7 µm) was supplied by Saint-Gobain Finland Oy, Isover, Finland, and MK (d_50_ ≤ 1.3 µm) was supplied by BASF Kaolin, Ludwigshafen, Germany. The chemical compositions of the precursors as determined using X-ray fluorescence (XRF, Oulu, Finland), and their densities, are listed in Table 1.

Two mix compositions were developed by two different SW/GW weight ratios of 5 and 1, while the metakaolin content was constant (40% of binder mass) for both mix compositions [37]. More details about the effects of using SW/GW ratios, alkaline activator properties, and sand/binder ratios can be found elsewhere [37]. These two mix compositions were selected based on the best performance in terms of mechanical strength and the leaching of heavy metals. In order to produce the mortars, standard sand was added with a sand-to-binder ratio of 1 (by weight). The sand used was “CEN-NORMSAND EN196-1” [38]. The sand size distribution varied from 0.08 to 2.00 mm and the maximum moisture content was 0.2%. The mix compositions were activated by a combination of liquid sodium silicate (Na_2_SiO_3_) (SiO_2_/Na_2_O = 2.5, containing ≈ 60% of water) and sodium hydroxide (NaOH) of 10 molar concentration as the alkali activator with a Na_2_SiO_3_/NaOH of 2.5. The alkali activator-to-binder ratio was fixed at 0.88 for all mix compositions. Sodium hydroxide was prepared by dissolving sodium hydroxide pellets in lab-controlled tap water (distilled water) 24 h before using and cooled down to room temperature (23 ± 1 °C).

The fiber-reinforced mix compositions were reinforced with 2% (of binder mass and almost 1.45% in volume) of PP fibers. The physical and mechanical properties of the PP fibers are listed in Table 2.

In order to introduce air voids to the mortars, the premade foam was used by different foam contents (25, 30, 35% of binder mass). The protein-based foaming agent used in this study was supplied by EABASSOC, Altrincham, England [39]. The foaming agent was added to water in a weight ratio of 1:33.3 and aerated to produce a stiff foam with a density of 45 kg/m^3^ [39]. The diameter of 80% of the bubbles was in the range of 0.3–1.5 mm [39]. The mix design details of the developed mix compositions are tabulated in Table 3.

### 2.2. Casting and Curing

In the batching process, dry ingredients (SW, GW, MK, and sand) were mixed for 3 min. Afterward, the sodium hydroxide and sodium silicate were mixed together for 3 min and then poured into the mixed dry ingredients, and mixtures were stirred for additional 3 min. PP fibers were gradually introduced to the mix compositions to avoid unfavorable balling impacts. It is worth mentioning that PP fibers were not added to the “Reference” mix compositions. Finally, the premade foam was poured into the mix compositions and mixed thoroughly until achieving homogenous mixtures. Then, the fresh mortar was cast into oiled molds with dimensions of 40 mm × 40 mm × 160 mm. All specimens were kept uncovered in the oven at a temperature of 60 °C for 24 h. After demolding, the specimens were cured in open air under lab-controlled conditions (45 ± 5% relative humidity (RH) and temperature of 23 ± 1 °C) for 28 days, until the testing day.

**Figure 1:**
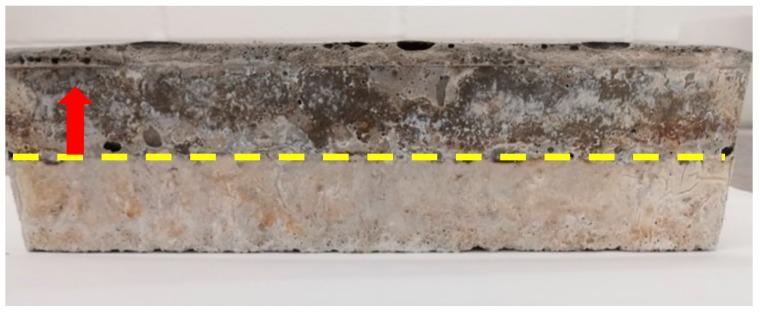
Separation of layers in the mix compositions with 25% foam content (without fibers).

During the casting trials, SW50/GW10 and SW30/GW30 lightweight mix compositions with 25% of foam content were cast without adding PP fibers (see Figure 1). The mixtures were cast by no settlement or dimension changes during the hardening phase. However, their densities were uncontrollable as four different densities were recorded as follows: 780, 1140, 1540, 1780 kg/m^3^ at the same foam content (25%). Moreover, notable separation of layers was observed. Interestingly, the same mix compositions and foam content were successfully cast when the PP fibers were added with no sign of layer separation. Therefore, the inclusion of PP fibers was found to be a critical component for providing homogeneity in the casting of SW50/GW10 and SW30/GW30 lightweight mortar. Therefore, it is postulated that the addition of PP fibers could increase the interactions of the matrix by attaching the matrix to the surface of PP fibers during the mixing process. As a result of the previous observations, all un-reinforced lightweight mix compositions were eliminated from this study.

### 2.3. Tests Procedures

#### 2.3.1. Mini-Slump Flow Test

The mini-slump measurements were carried out to assess the differences in the workability of both fiber-reinforced un-foamed mix compositions (SW50/GW10 and SW30/GW30). The flowability of the fiber-reinforced un-foamed mortars was evaluated according to ASTM C1437 recommendations [41]. Two orthogonal diameters of the spread compositions were measured, and the average diameter was registered. The mini-slump measurements were repeated at 0, 15, 30, 60 and 120 min to assess the workability loss with time while the fresh fiber-reinforced mortars were kept in sealed containers in lab-controlled conditions (23 ± 1 °C).

#### 2.3.2. Density Measurement

Each specimen was weighed and its dimensions were measured with a digital caliper. The dry density of each specimen was calculated by dividing the mass by the apparent volume. The reported density of each mix composition is the average of three samples measurements.

#### 2.3.3. Ultrasonic Pulse Velocity

**Figure 2:**
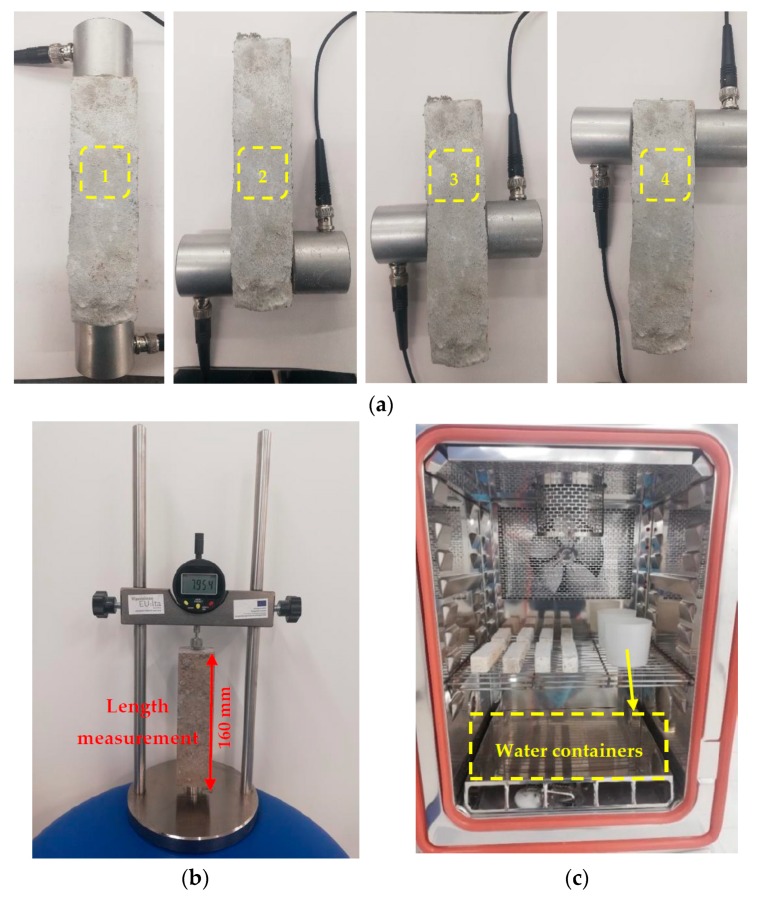

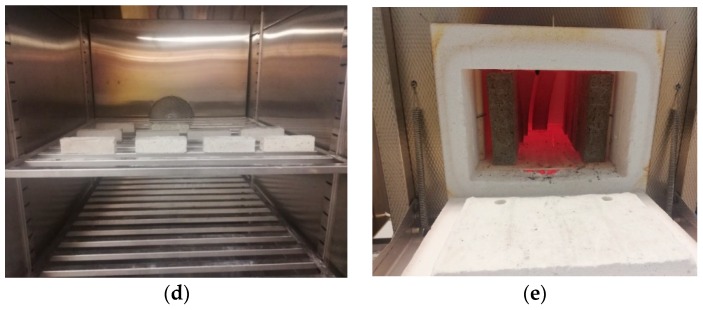
(**a**) UPV measuring at different positions; (**b**) test device used for the drying shrinkage measurements; (**c**) freeze/thaw test; (**d**) carbonation chamber; (**e**) oven used for the high-temperature test.

A non-destructive ultrasonic pulse velocity tester (model no.: C369N), Italy, with measuring range (0–3000 µs) and accuracy (±0.1 µs) was used to assess the quality of the mortar and the homogeneity of air voids distribution. Two 55 kHz transducers with an accuracy of ±2% for distance and ±1% for travel time and were adopted. During the sample preparation, Vaseline was used to fit the surfaces of the transducer to the sample surface tightly. The UPV test evaluates the quality of the mortar and the presence of air voids by measuring the velocity of an ultrasonic wave traversing the mortar between two transducers (see Figure 2a). A higher velocity indicates a denser structure of the mortar with lower air voids. Adding foam and fiber for the mortar mixtures affects the porosity of the mortar. Therefore, to evaluate the impact of the foam contents and fiber additions on the compactness of the samples, the UPV test was used according to ASTM C597 recommendations [42]. The pulse velocity was measured by Equation (1).

(1) $$V=\frac{L}{T}$$

Where V is the pulse velocity (m/s), L is the distance between two transducers (m) and T is the transmission time(s).

#### 2.3.4. Compressive Strength

To investigate the strength of mix compositions under compression loading, the two broken portions that resulted from the flexural test were used, according to ASTM C349 recommendations [43]. All specimens were tested under a compressive load with a constant displacement rate of 1.8 mm/min using Zwick Roell (Z100), Ulm, Germany, testing machine with a hydraulic press of 100 kN static loading capacity. The compressive strength of each mix composition was obtained by averaging the values of six portions.

#### 2.3.5. Flexural Strength

Flexural strength was assessed under three point bending (TPB) loading, according to ASTM C78 recommendations [44]. Three prismatic beams for each mix composition were tested using Zwick Roell (Z100), Germany, under flexural loading with a deflection rate of 0.6 mm/min to assess the effects of foam contents and fiber reinforcement on flexural performance. Flexural strength (Fr) was calculated using Equation (2)

(2) $$\mathrm{Fr}=\frac{1.5\;\mathrm{FL}}{{\mathrm{bh}}^{2}}$$

where F is flexural load (N), L is the span length (120 mm), b and h are the width (40 mm) and height (40 mm) of the prismatic beams.

#### 2.3.6. Drying Shrinkage

In total, 20 prismatic beams (two beams/mix composition) with dimensions of 40 mm × 40 mm × 160 mm were prepared using the same procedures and curing conditions as discussed earlier to measure the ASTM C157-recommended drying shrinkage rate [45] (see Figure 2b). 24 h after demolding, the first measurement was taken. The rest were taken daily during the first week, then twice a week thereafter until the stabilization of the length was achieved and no further reduction in length was recorded.

#### 2.3.7. Efflorescence Assessment

To monitor the efflorescence of the mix compositions and the effects of adding the foam contents and fiber, the 28-day-old specimens were placed in 20 mm (half of the specimen height) of water for seven days in laboratory conditions (45 ± 5% RH and temperature of 23 ± 1 °C). Visual observations were captured. The level of water was kept constant throughout the observation period.

#### 2.3.8. Water Absorption and Permeable Porosity

The effects of different foam contents on permeable porosity and water absorption were assessed according to ASTM C642 recommendations [46]. Three specimens from each mix composition were totally immersed in a water tank with an average water temperature of 20 ± 2 °C for 24 h. Thereafter, the buoyant mass of each water-saturated specimen was measured while suspending the specimen by a thin wire completely submerged in water (M_B_: Buoyant mass). Then, the specimen was removed from the water and, by using a wet cloth, the observable surface water was removed, and the mass of dry surface water-saturated specimen was registered (M_S_). Subsequently, the specimens were placed in the oven at 105 °C and the first oven-dry mass (M_D_) was measured after 24 h. However, the oven-dry mass of each specimen was also measured every two hours after the first measurement until the increment of the mass loss was not greater than 0.1% of the last measured mass. Permeable porosity and water absorption were calculated by Equations (3) and (4), respectively.

(3) $$\mathrm{Permeable}\;\mathrm{porosity}\;\left(\%\right)=\left(\frac{M_{S}-M_{D}}{M_{S}-M_{B}}\right)\times 100$$

$$\mathrm{Water}\;\mathrm{absorption}\;\left(\%\right)=\left(\frac{M_{S}-M_{D}}{M_{S}-M_{B}}\right)\times 100$$

#### 2.3.9. Freeze and Thaw Resistance

**Figure 3:**
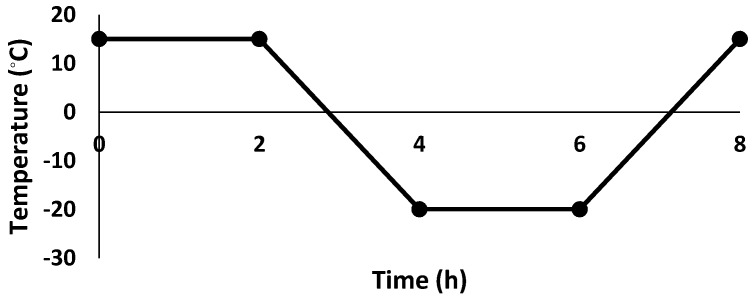
Adopted protocol for temperature variations per one cycle in freeze/thaw test.

At the age of 28 days, the freeze/thaw assessment was executed on the specimens. In order to simulate the real-life conditions where the lightweight mortar is usually used indoors, and hence could be subjected to humidity variations rather than direct contact with water, three specimens from each mixture were kept beside a water container in the freeze-and thaw-machine (see Figure 2c). The test temperatures ranged from −20 °C to 15 °C; test procedures were followed based on [47]. Due to the freeze/thaw cycles, water in the tanks will have been evaporated, increasing the humidity in the chamber. The tested specimens will have absorbed this humidity. The specimens were kept for two hours at 15 °C and two hours at −20 °C, while decreasing the temperature from 15 °C to −20 °C took two hours. Similarly, the chamber temperature increased from −20 °C to 15 °C over the course of two hours. Figure 3 depicts one cycle. The test consisted of three cycles per 24 h. The density and UPV of each specimen were measured before the test and after 60 freeze/thaw cycles. In addition, after 60 cycles the specimens were tested under flexural and compressive loadings.

#### 2.3.10. Carbonation Test

To analyze the effects of carbonation on the mix compositions, 28-day-old cured specimens (three specimens/each mix composition) were exposed to carbonation for seven days with 5% CO_2_ concentration, 60% RH, and the temperature of 23 °C (see Figure 2d). The density and UPV of the specimens were measured before and after exposure to the carbonation test to evaluate the changes that occurred due to CO_2_ absorption. Then, the carbonated prismatic beams were tested under both flexural and compressive loadings to assess the effect of carbonation on mechanical properties.

#### 2.3.11. High-Temperature Test

To assess the impact of high temperatures on the mechanical properties and quality of the mix compositions, three samples for each mix composition were heated in the oven under high temperatures of 200, 350, and 500 °C at the age of 28 days (see Figure 2e). The oven took three hours to reach the test temperature and the temperature was kept constant for four hours. Then, the specimens were kept inside the oven to cool down to room temperature. The density and UPV were measured before and after exposure to high temperatures in order to compare the changes that resulted from heating the specimens under high temperatures. The compressive and flexural strengths of the heated specimens were then investigated.

#### 2.3.12. X-Ray Micro-Computed Tomography Technique

**Figure 4:**
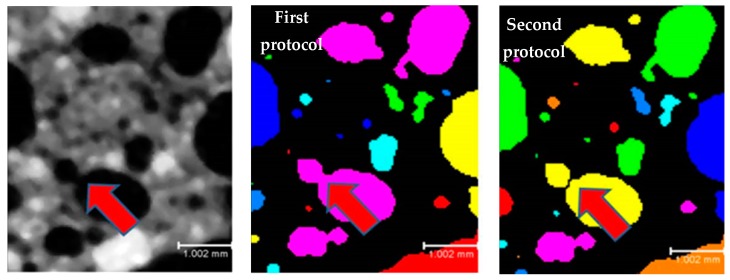
Numbers of pores measuring protocols.

X-ray micro-CT was used to analyze the internal porous structure of the lightweight mix compositions [48]. Four specimens for each mix composition were assessed after being exposed to different conditions (ambient, carbonation, freeze/thaw, and high-temperature). X-ray micro-CT scanning was executed by using an XRadia CT-400 tomography device (XRadia, Concord, CA, USA) with a resolution of 37 µm for 1 pixel was adopted. The beam energy and the intensity were set to 80 kV and 7 W, respectively. During this test, 1600 projection images at an exposure time of two seconds per projection were captured through the charge-coupled device (CCD) camera. Modeling of the 3D internal pore structure of the samples, estimation of the total porosity, and the pore numbers and size distribution were executed by using Avizo Fire 3D-image analysis software (Thermo Scientific™ Avizo™ Software, Thermo Fisher Scientific, Waltham, MA, USA) [49]. Regarding the measurements of the numbers of pores, the first protocol was adopted where the connected air voids were measured as one pore (see Figure 4). In addition, segmentation of pore sizes was performed via histograms.

## 3. Results and Discussion

### 3.1. Mini-Flow Slump Test

**Figure 5:**
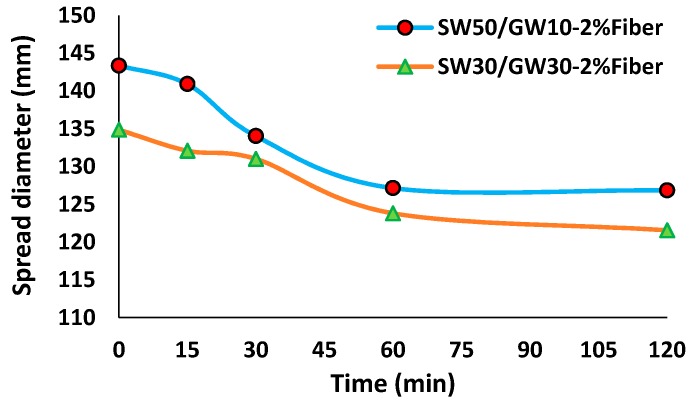
Slump diameter of the fiber-reinforced un-foamed mortars.

The SW50/GW10-2% Fiber mix composition showed a higher slump diameter and workability, compared to SW30/GW30-2% Fiber (see Figure 5). Hajimohammadi et al. [50] detected an inversely proportional relationship between the hardened densities of the lightweight slag-based AAM and its paste workability. They illustrated that, at the same foam content, the mix composition with higher paste workability revealed a lower density, as compared to one with lower paste workability. The lower workability of the SW30/GW30 mix composition can explain its higher hardened density (discussed later in Section 3.2.1). This characteristic is attributed to the higher shear stress on the air bubbles that were introduced during the mixing process and setting times, which result in a higher rate of collapsed pores, as compared to the SW50/GW10 mix composition. On the other hand, the higher workability of the SW50/GW10 mix composition can permit merging of the introduced air bubbles, resulting in larger air voids and a lower density.

### 3.2. Hardened State Properties

#### 3.2.1. UPV and Density

**Figure 6:**
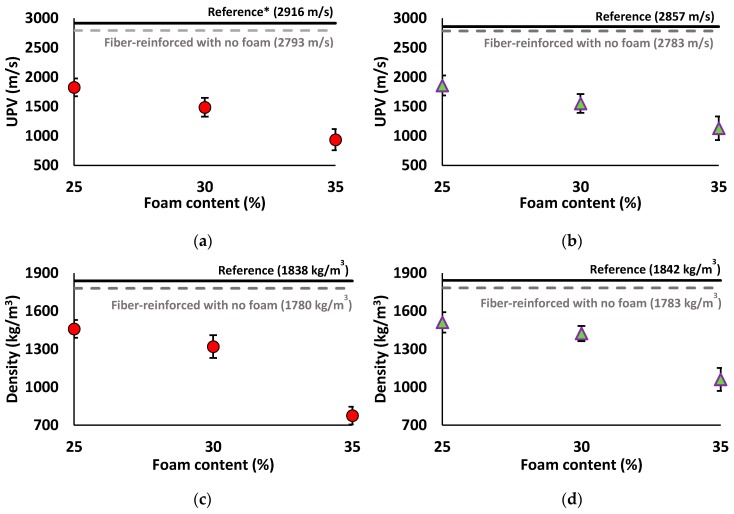
Impacts of adding PP fibers and different foam contents on the UPV results of mix compositions: (**a**) SW50/GW10; (**b**) SW30/GW30; and density of mix compositions: (**c**) SW50/GW10; (**d**) SW30/GW30. (* plain mix composition with no foam and fiber addition).

Figure 6 depicts the effects of introducing PP fibers and different foam contents on the UPVs and densities of the mix compositions. Based on the results, the addition of PP fiber reduced the UPV and density of both mix compositions, as compared to the reference mixtures. Around 5% (2793 m/s) and 2.5% (2783 m/s) reductions in the UPV due to the addition of the PP fiber was recorded in the SW50/GW10-2% Fiber and SW30/GW30-2% Fiber mix compositions, respectively, with respect to the reference mix compositions. Moreover, fiber inclusion showed about a 3% (≈1780 kg/m^3^) reduction in density as compared to the reference mixtures (≈1840 kg/m^3^). The measured reductions in the UPV and density with the addition of the fiber were also reported in [51] and referred to the increase in air voids inside the mixtures.

Regarding the lightweight mix compositions, a steady reduction in the UPV and density when increasing the foam content was observed. Introducing foam to the mix compositions resulted in a density in a range of (770–1460 kg/m^3^) and (1060–1510 kg/m^3^) for mix compositions with SW/GW ratios of 5 and 1, respectively. With 35% foam content, the density reduction with SW/GW ratio of 5 was 55% (resulting in 770 kg/m^3^) and at SW/GW ratio of 1 the density reduction was 40% (resulting in 1060 kg/m^3^). The UPV of the lightweight mix compositions was inversely proportional to the foam content because of the higher amount of air voids introduced with higher foam content. Therefore, the maximum reductions in the UPV were recorded at the mix compositions containing 35% foam content in which 65% and 60% reductions were registered in the SW/GW ratios of 5 and 1, respectively compared to their normal weight fiber-reinforced mortars. On the other hand, the minimum UPV (density) reductions of around 35% (18%) and 33% (15%) were detected at 25% foam content with SW/GW ratios of 5 and 1, respectively, comparing with SW50/GW10-2% Fiber and SW30/GW30-2% Fiber mix compositions. It is worth mentioning that error values presented in all figures are equal to (2×standard deviation).

**Figure A1:**
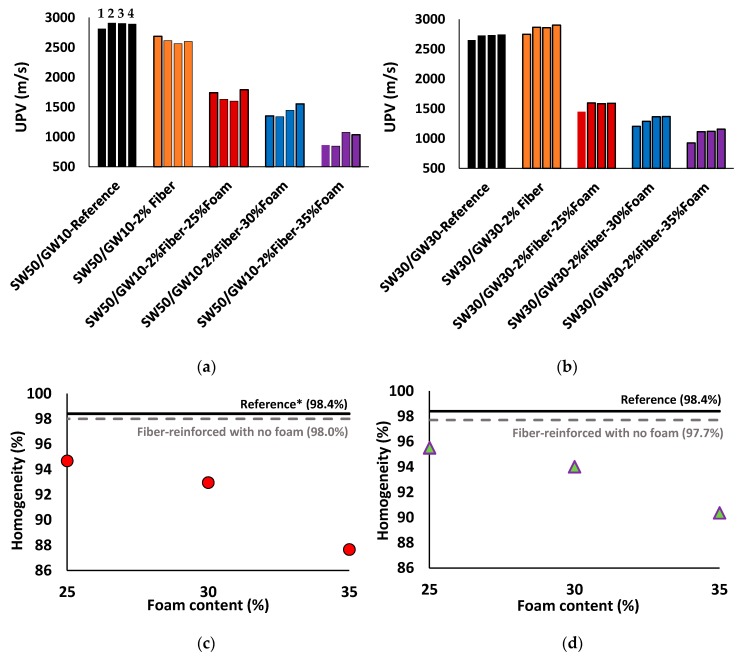
Effects of foam content and fiber addition on the UPV measurements at four different positions of prismatic samples: (**a**) SW50/GW10; (**b**) SW30/GW30; and on the homogeneity of UPV measurements of the mix compositions: (**c**) SW50/GW10; (**d**) SW30/GW30. (* plain mix composition with no foam and fiber addition).

Assessing the homogeneity of the fiber distribution in the fiber-reinforced mortars is crucial to ensuring that the fibers were well-distributed and the effect of fibers balling in the samples were minimized. Similarly, Hajimohammadi et al. [50] illustrated the importance of assessing the homogeneity of pores distribution in the lightweight AAM porous structure. It was reported that specimens with higher pore distribution homogeneity exhibited higher strength. Therefore, to assess the pore distribution homogeneity of the fibers and air voids in the mix composition structure, UPV measurements were taken in four different positions, as shown in Appendix A. Regarding UPV measurements (see Figure A1a,b in Appendix A), a fluctuation in the UPV results was detected. The homogeneity percentage was calculated through the standard deviation and coefficient of variation (standard deviation/mean) of the UPV measurements at the four different positions. Normal weight mix compositions (reference and fiber-reinforced) recorded UPV homogeneity of >97% regardless of the SW/GW ratio (see Figure A1c,d in Appendix A). Therefore, it was concluded that the PP fibers were homogenously distributed within the specimens. Furthermore, homogeneity was decreased with the addition of foam to the mix compositions. Homogeneity of the UPV measurements was inversely proportional to the foam content. Regarding the lightweight mix compositions, the highest UPV homogeneities of around 95% were achieved at foam content of 25%, regardless of the SW/GW ratio. The lowest homogeneities (≈88 and 90%) were measured in mix compositions with 35% foam content and SW/GW ratios of 5 and 1, respectively. The minimum homogeneities detected at the 35% foam content can be attributed to the higher water content introduced to the mix compositions by the added foam, and therefore higher workability. It was reported in [51] that mix compositions with the highest workability resulted in increasing the extent of air voids rearrangement, and therefore the lowest homogeneity of pore distribution.

#### 3.2.2. Strength Assessment

**Figure 7:**
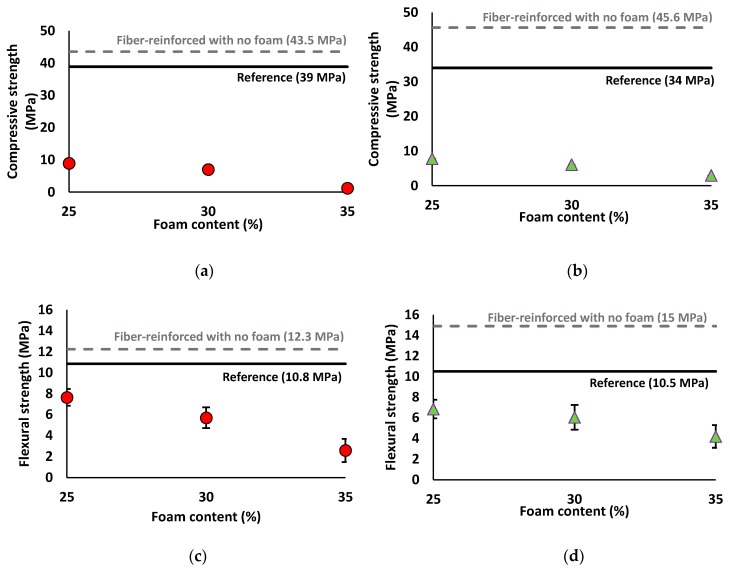
Effects of adding PP fibers and different foam content on the compressive strength of mix compositions: (**a**) SW50/GW10; (**b**) SW30/GW30; and flexural strength of mix compositions: (**c**) SW50/GW10; (**d**) SW30/GW30.

The impact of adding PP fiber on the compressive and flexural strengths of the mix compositions are shown in Figure 7. Despite the higher porosity of the fiber-reinforced mixture as assessed by the UPV results, the PP fibers increased the compressive and flexural strength of the mix compositions. The fiber inclusion helped in limiting crack propagation under the compression load [51]. Moreover, the bridging action of PP fibers can explain the increase in flexural strength [35].

According to the results, the compressive and flexural strength gain due to PP fiber inclusion was more significant in the mix composition developed with SW/GW:1. 35% (46 MPa) and 36% (15 MPa) increases in the compressive and flexural strengths were measured, respectively, in the SW30/GW30-2% Fiber, compared to SW30/GW30-Reference. However, the SW50/GW10-2% Fiber mix composition (41 MPa) showed a 5% increase in compressive strength, as compared to SW50/GW10-Reference (39 MPa). In addition, an increase of around 18% (≈13 MPa) in flexural strength was observed in the SW50/GW10-2% Fiber, with respect to SW50/GW10-Reference (≈11 MPa).

Considering the lightweight mix compositions, it was revealed that increasing the foam content significantly reduced the compressive and flexural strengths. The inverse relationship between foam content and compressive and flexural strengths was also reported in [1,52,53,54]. In line with UPV and density results, increasing the foam content was accompanied by higher reductions in the compressive and flexural strengths. Therefore, the maximum compressive (flexural) strengths of the lightweight mix compositions of around 9 (8) and 8 (7) MPa were assigned for the SW50/GW10-2% Fiber-25% Foam and SW30/GW30-2% Fiber-25% Foam, respectively. The minimum compressive (flexural) strengths of 1.2 (2.6) and 3 (4.2) MPa were measured in the SW50/GW10-2% Fiber-35% Foam and SW30/GW30-2% Fiber-35% Foam, respectively. However, it was detected that the flexural strength reduction due to the increase of foam content from 25% to 35% was governed by the SW/GW ratios and foam contents. The strength reduction in SW50/GW10 (strength loss of ≈5 MPa) was more significant than SW30/GW30 (strength loss of ≈2.5 MPa). The reason for significant or slight flexural strength loss could be related to the differences in the pore structure of the lightweight mix compositions. It will be shown and discussed using CT scanning technique in Section 3.4 that the SW/GW ratios and foam contents significantly affect the total porosity and pore volumes. Therefore, different strength loss was observed in both lightweight mix compositions.

Compared to the findings in [50], where the specimens with a density of 750 kg/m^3^ for the lightweight slag-based AAM achieved a normalized strength (average strength/average density) of about 0.6 kN·m/kg, a higher normalized strength of around 1.6 kN·m/kg was detected in SW50/GW10-2% Fiber-35% Foam (≈775 kg/m^3^). Moreover, it was stated in [15,55,56] that the flexural strength-to-compressive strength ratio of the lightweight cementitious compositions is in the range of 0.1–0.58, while in this study a ratio of (0.8–2.2) was recorded, which can be attributed to the inclusion of the PP fiber. Zollo et al. [57,58] demonstrated the significant improvement of the mechanical properties of the lightweight OPC-based concrete reinforced with the PP fiber. Similarly, Bing et al. [59] reported about a 30% increase in the flexural strength of the lightweight OPC concrete with the inclusion of PP fiber compared to the plain specimens.

It is worth stating that the low error values were found for the compressive strength of all mix compositions, therefore, these error bars are not easily visible in Figure 7a,b.

**Figure A2:**
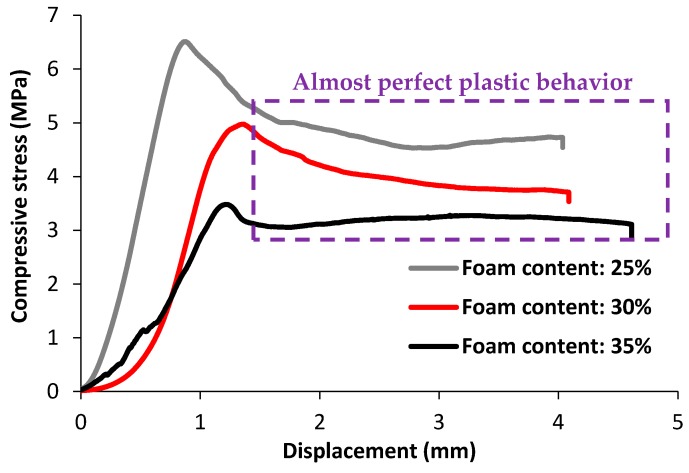
Compressive stress versus displacement curves of the developed lightweight AAM with different foam contents.

Interestingly, while applying the compressive load on the specimens, an almost perfect plastic behavior was observed after entering the nonlinear phase (see Figure A2 in Appendix A). Similar behavior was also noticed in [60], where the authors attributed the behavior to the internal pore structure rearrangement and the collapse of the pores. By increasing the foam content, this nonlinear contribution became much more pronounced.

**Figure 8:**
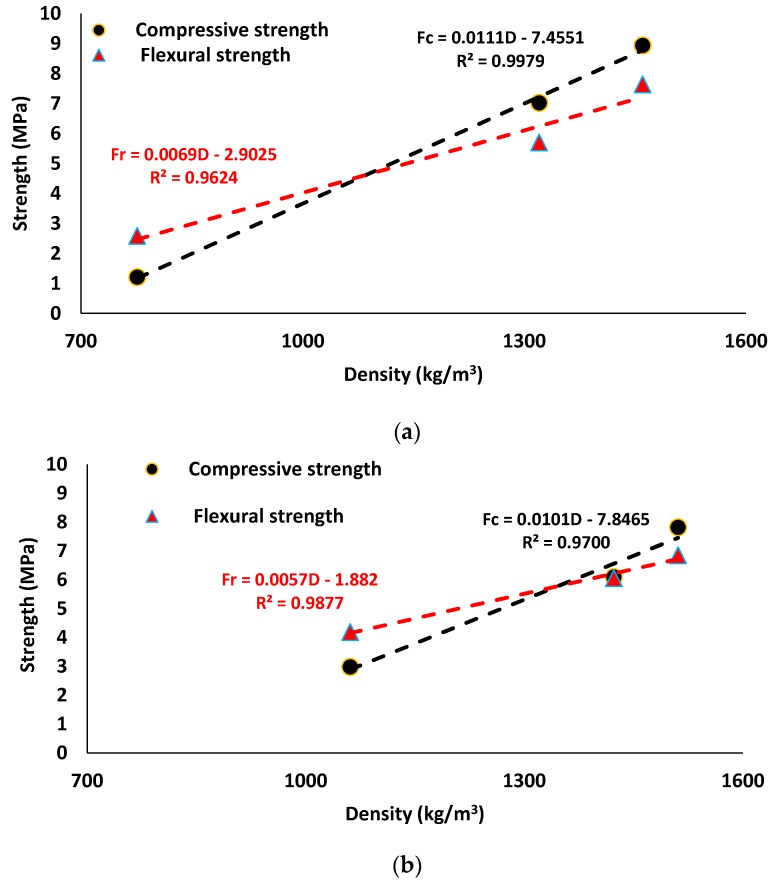
Correlation between the compressive and flexural strengths and density of lightweight mix compositions: (**a**) SW50/GW10; (**b**) SW30/GW30.

Figure 8 represents the correlation between strengths (where, Fr and Fc are flexural and compressive strengths, respectively) and densities (D) of the lightweight mineral wool-based alkali activated mortar using linear regression. Both mix compositions showed linear relationships with a high coefficient of determination (R^2^ ≥ 0.96). According to the developed linear relationships, it was detected that the increasing rates of strength due to the increase of density was higher in the SW50/GW10 mix composition when compared to the SW30/GW30. Moreover, it was noticed that the reductions in the flexural strength due to the reduction in density had a lower rate than the measured reductions in the compressive strength. Similar findings were found in [52,55,61]. However, Abdollahnejad et al. [15] indicated that no correlation between compressive strength and density in lightweight FA-based AAM could be established.

#### 3.2.3. Drying Shrinkage

**Figure 9:**
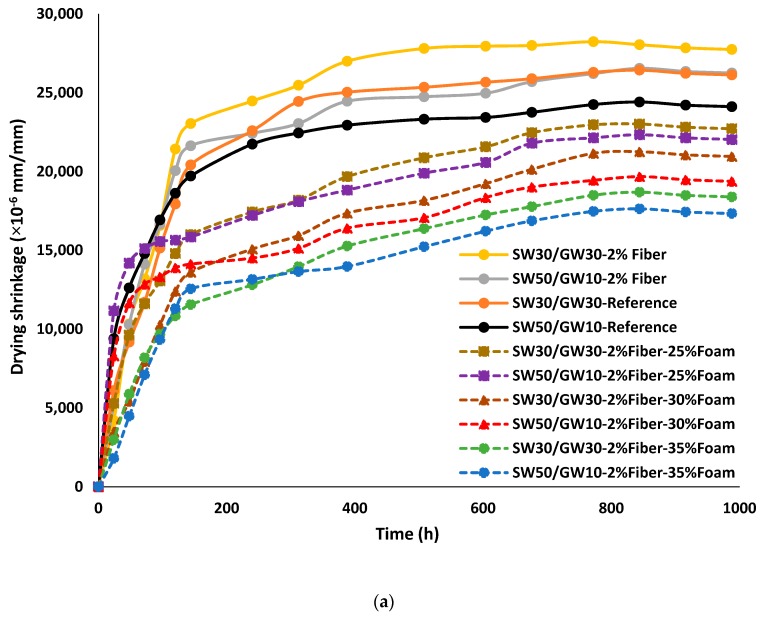

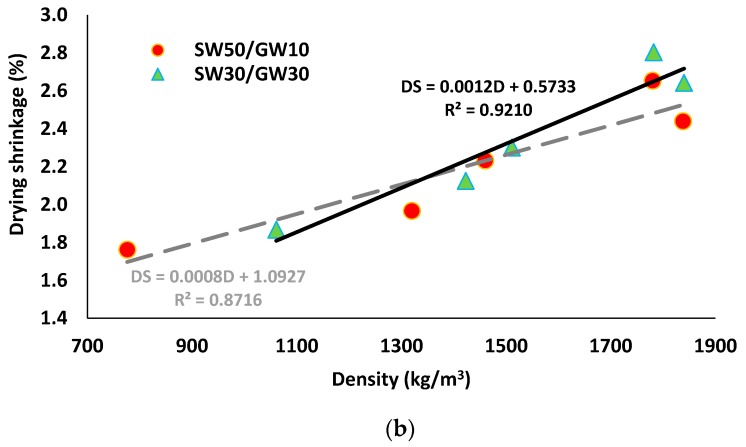
(**a**) The impact of adding PP fibers and different foam contents on the drying shrinkage (mm/mm) of mix compositions; (**b**) the relationship between the drying shrinkage and density of mix compositions.

The effects of adding different foam contents and introducing PP fiber on the drying shrinkage of the mix compositions are compared in Figure 9a. Based on the results obtained, it was revealed that the length stabilization was measured for all mix compositions after ≈1000 h. Nevertheless, normal weight mix compositions reached around 90% of their maximum drying shrinkage after around 315 h (about 14 days), while this percentage was recorded after 605 h (almost 26 days) in the case of lightweight mix compositions (see Figure 9a). In addition, no cracks were visually observed on the surface of the tested specimens during the measurement period.

According to the drying shrinkage measurements, it was observed that increasing the SW/GW resulted in decreasing the drying shrinkage of the mix compositions. Drying shrinkage of around 2.4% and 2.6% were measured at SW50/GW10-Reference and SW30/GW30-Reference, respectively, as compared to their initial lengths (see Figure 9a). Introduction of PP fiber has negative impacts on the drying shrinkage, regardless of SW/GW ratio. The fiber-reinforced mixtures (SW50/GW10-2% Fiber and SW30/GW30-2% Fiber) registered the maximum drying shrinkage of around 2.6% and 2.8%, respectively. Despite the proposed benefits defined in [62,63] for using fibers in mitigate the negative impacts of drying shrinkage in AAM, the aim of adding PP fibers in this work was to provide uniformity of the foamed mortar, while separation between layers was observed in the plain foamed mortar, as shown in Figure 1.

The lightweight mix compositions achieved a lower drying shrinkage (1.7–2.3%), as compared to fiber-reinforced normal-weight (no foam) mix compositions. Drying shrinkage of the developed lightweight mix compositions was inversely proportional to the foam content, regardless of SW and GW contents. Similarly, the inverse relationship between foam content and drying shrinkage was noticed in [14,64]. In contrast, the drying shrinkage of alkali-activated slag based foam concrete was directly proportional to the foam content in [52]. Regarding the SW50/GW10 mix composition, a drying shrinkage of around 2.2, 1.9, and 1.7% was recorded for the specimens fabricated with 25, 30, and 35% foam content, respectively. On the other hand, about 2.3, 2.1, 1.9% of drying shrinkage were measured at SW30/GW30 specimens with 25, 30, and 35% foam content, respectively. Therefore, the minimum drying shrinkage of around 1.7% and 1.9% were achieved by SW50/GW10 and SW30/GW30 mix compositions, respectively, with 35% foam content.

Figure 9b clarifies the linear relationships between the drying shrinkage (DS) and density (D) of the mix compositions. Compared to the fiber-reinforced normal-weight mix compositions, around 35% reduction in the drying shrinkage was achieved in both mix compositions with 35% foam content. The lower drying shrinkage of the lightweight mortar can be attributed to its lower paste content. It was mentioned in [65] that mixtures with lower paste content have a lower negative effect on the drying shrinkage, which explains the continuous reduction trend of the drying shrinkage with increased foam content. Mix compositions with higher foam content, most likely, have larger pores with thinner pore walls and therefore lower overall paste content. Moreover, Hajimohammadi et al. [21] showed that the drying shrinkage of the lightweight AAM follows a similar trend of their paste and mortar with the relatively lower extent of drying shrinkage. Their findings are confirmed in this study, so that lightweight SW30/G30 exhibited a higher drying shrinkage than SW50/GW10, regardless of the foam content, which is similar to the normal-weight mix compositions trend.

#### 3.2.4. Efflorescence Assessment

**Figure 10:**
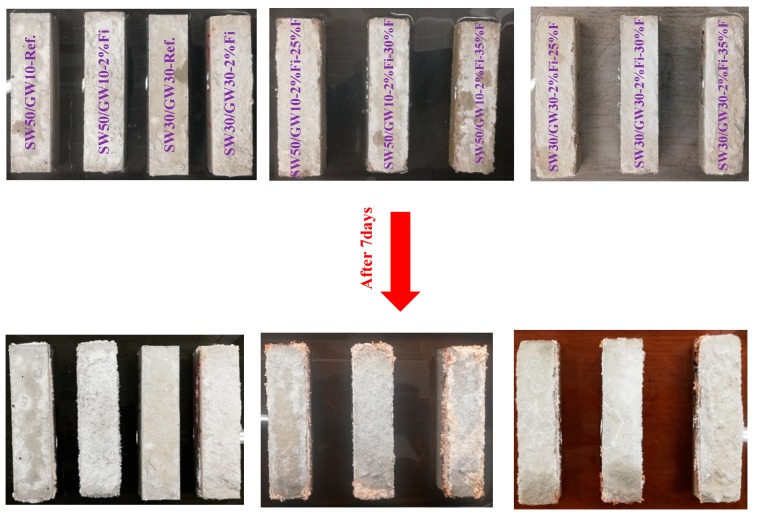
Visual observations of the efflorescence. (Ref., Fi, and F were used in place of Reference, Fiber, and Foam, respectively.).

The efflorescence rate of each mix composition after seven days was visually monitored. The visual observations are shown in Figure 10. After seven days, the lightweight mix compositions showed a higher efflorescence than the normal-weight mix compositions. Moreover, it was noticed that increasing the SW content increased the efflorescence for all mix compositions. The addition of PP fibers also increased the efflorescence rate, regardless of the SW/GW ratio, as compared to reference mix compositions. The visual observations revealed that efflorescence increased with increased foam content, regardless of SW and GW content. As a result, mix compositions with 35% foam content showed the highest efflorescence. This phenomenon is due to the higher porosity in the specimens with higher foam content, which led to higher water penetration and release of alkali cations [1,18]. However, the efflorescence observations of this study conflict with Zhang et al. [66] and Şahin et al. [55]. In [66], fly ash-based foamed AAM was monitored to investigate the effects of the pore structure on the efflorescence. It was observed that specimens with higher porosity have lower efflorescence products on the surface of the specimens after seven days. Nevertheless, in the same study, the efflorescence products were detected by optical microscopy, and it was observed that the efflorescence products were accumulated and trapped inside the macro-pores. Similarly, Şahin et al. [55] explained the lower efflorescence of lightweight AAM by its lower solid content, which resulted in a reduction of leachable alkali solution. Furthermore, they stated that the higher porosity and pore size would limit the leaching of the alkali solution.

As aforesaid, the developed lightweight mix compositions could be used for indoor panels. Relative humidity in indoor environments could be low or the panels would not be directly exposed to the water contact, therefore, efflorescence problem may not be crucial in this case.

#### 3.2.5. Water Absorption and Permeable Porosity

Introducing PP fiber showed insignificant impact on water absorption and permeable porosity compared to the reference mixtures. The lightweight mortar exhibited higher water absorption (≈24–47%) and permeable porosity (≈31–42%) than the normal weight mix composition: ≈14% and ≈21%, respectively. Water absorption and permeable porosity were increased significantly with decreasing density (or increasing the foam content). Similar findings were found in [55,67]. Since the results conflict with Nambiar et al. [68], who observed a reduction in water absorption with a decreased density of OPC/FA-based foam concrete. However, Petlitckaia et al. [69] proved that increasing the foam content resulted in a rising open porosity of the lightweight MK-based AAM. In addition, Masi et al. [70] claimed that water absorption is dependent on the open porosity and connectivity between the pores rather than the total porosity of the lightweight FA-based AAM. A similar relation was also noticed in this study, as water absorption and permeable porosity were linearly correlated with density, therefore water absorption and permeable porosity are linearly correlated to each other.

The maximum water absorptions of around 46% and 39% were recorded at SW50/GW10 and SW30/GW30 mix compositions with 35% foam content, respectively. Yang et al. [67] reported a higher water absorption (≈90%) of the lightweight slag-based AAM (700 kg/m^3^) using preformed air bubbles. The maximum permeable porosity levels (≈42% and 38%) were measured at SW50/GW10 and SW30/GW30 mix compositions with 35% foam content, respectively. However, the minimum water absorption (≈25%) and permeable porosity (≈32%) were achieved in lightweight mortar with 25% foam content, regardless of SW and GW content.

**Figure 11:**
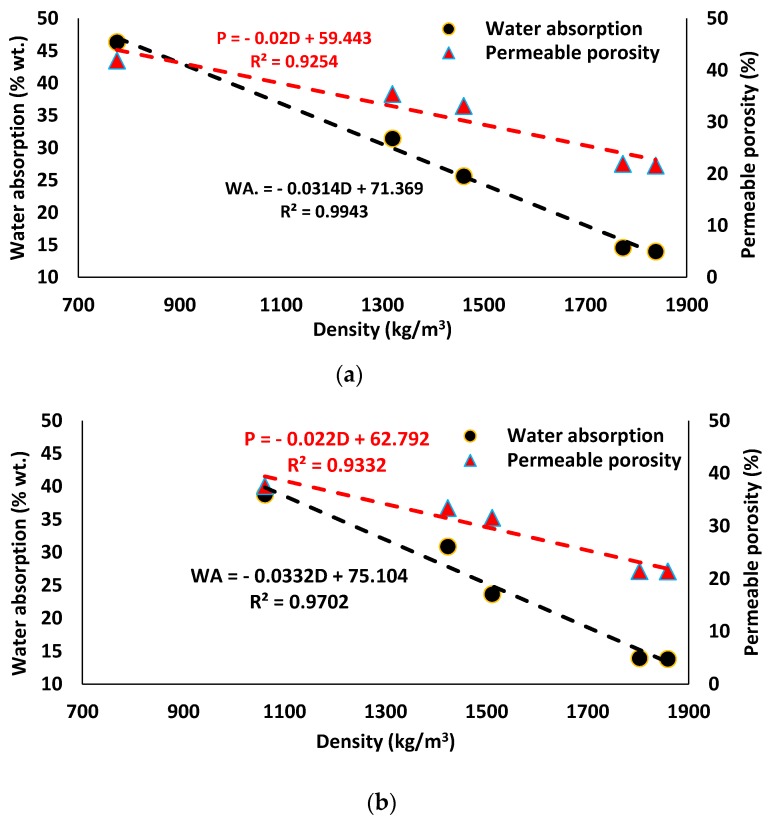
The relationship between water absorption, permeable porosity, and density of mix compositions: (**a**) SW50/GW10; (**b**) SW30/GW30.

The water absorption (WA) (expressed by the increase in mass as a percentage of the pre-test dry mass) and permeable porosity (P) of the normal weight and lightweight mortar were plotted as a function of density (D) in Figure 11. According to the results, water absorption and permeable porosity of both mix compositions revealed linear inverse relationships with the density accompanied with high coefficients of determination (R^2^ ≥ 0.97) and (R^2^ ≈ 0.93), respectively.

### 3.3. Effects of Harsh Conditions on Hardened Properties

#### 3.3.1. Carbonation Resistance

**Figure 12:**
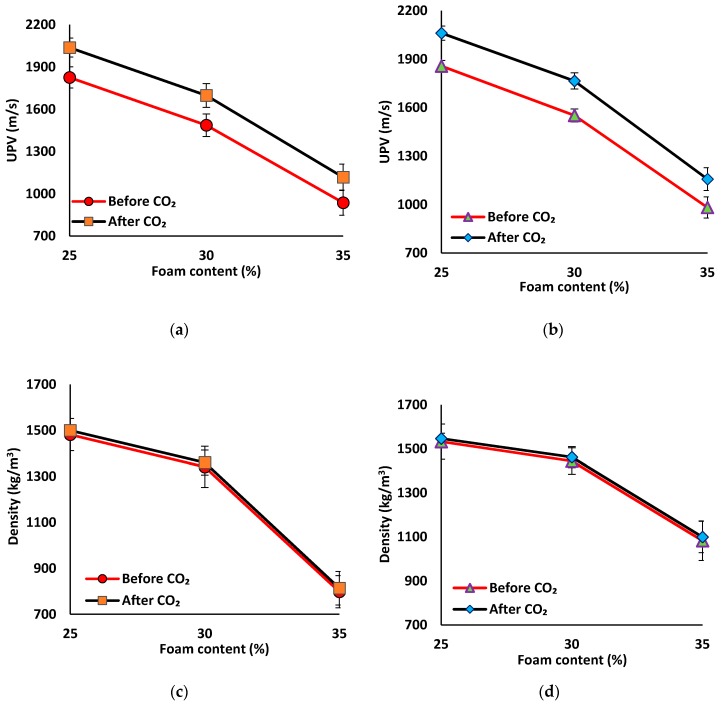
Effects of carbonation on the UPV results of mix compositions: (**a**) SW50/GW10; (**b**) SW30/GW30; and density of mix compositions: (**c**) SW50/GW10; (**d**) SW30/GW30.

The impact of carbonation on the UPV and density of lightweight mix compositions were compared in Figure 12. The measured UPV and density for the normal-weight mix compositions were reported in Table A1, Appendix A. Exposing the specimens to 5% CO_2_ gas flow under a pressure of one bar for seven days resulted in increasing the UPV and density for all mix compositions. The measured increases can be referred to the formed crystals (mainly calcium carbonate (Calcite) and sodium carbonate) due to the reaction between calcium and sodium sources and absorbed CO_2_ gas [12]. The increase of the UPV and density after carbonation were inversely proportional to the density of the mix compositions so that lightweight mix compositions had higher increases in UPV and density compared to the normal weight mix compositions.

The density gains measured at all carbonated mix compositions were limited, which is ≈0.5% in case of reference and fiber-reinforced normal-weight mix compositions. An increase of around 1.5% in density was recorded in the lightweight mix compositions. However, a slight increase in density gain was detected when increasing the foam content in the lightweight mix compositions. Additionally, about a 3% increase in the UPV was registered in normal-weight mix compositions with respect to uncarbonated specimens. The increase in the UPV was higher in the lightweight mix compositions, where 11–20% increases were detected, as compared to the uncarbonated state. Similar to the density, the increase in the UPV was higher in the mix composition with higher foam content. The maximum increase in the UPV of around 20% was achieved in the lightweight mortar with 35% of foam content, compared to the uncarbonated counterparts. An increase of around 12% in UPV was measured in specimens with 25% foam content.

**Figure 13:**
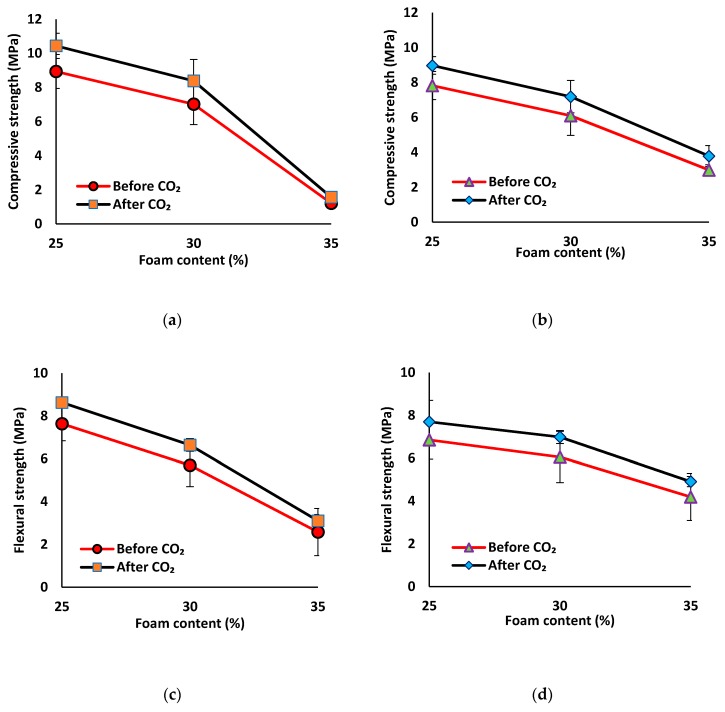
Impacts of the carbonation on the compressive strength of mix compositions: (**a**) SW50/GW10; (**b**) SW30/GW30; and on the flexural strength of mix compositions: (**c**) SW50/GW10; (**d**) SW30/GW30.

According to the results, all carbonated mix compositions showed higher compressive and flexural strengths as compared to the uncarbonated condition (see Figure 13, and Table A1 in Appendix A). The lightweight mortars gained a higher increase (≈15–30%) in the compressive and flexural strengths than the normal-weight mix compositions (≈10–14%). The minimum increase in the compressive and flexural strengths (≈10%) due to carbonation was detected in the reference mix compositions. Introduction of PP fibers resulted in increasing compressive and flexural strength gain up to 14%. The higher compressive and flexural strength increase in the fiber-reinforced mix compositions can be attributed to increasing of the friction between the fiber and matrix due to the formed crystals, as well as to the air voids in the skeleton [12].

Similar to the UPV and density, the increases in the compressive and flexural strengths due to carbonation were directly proportional to the foam content level. Jones and McCarthy [31,32] showed that low-density concrete was carbonated at a higher rate under accelerated carbonation. Moreover, Chica et al. [33] indicated that a higher open porosity increased the carbonation rate. The open porosity can facilitate CO_2_ penetration into the internal structure of the specimens. As it was shown in (Section 3.2.5), increasing the foam content (lower density) resulted in higher open and permeable porosity, and hence, more absorbed CO_2_ gas and consumption of sodium sources, resulting in increasing the formation of the crystals. Therefore, the maximum increases in the compressive and flexural strengths of around 30% and 20%, respectively, were detected at 35% foam content, as compared to the un-carbonated state. An increase of around 15% in the compressive and flexural strengths was measured at 25% foam content.

#### 3.3.2. Freeze and Thaw Resistance

**Figure 14:**
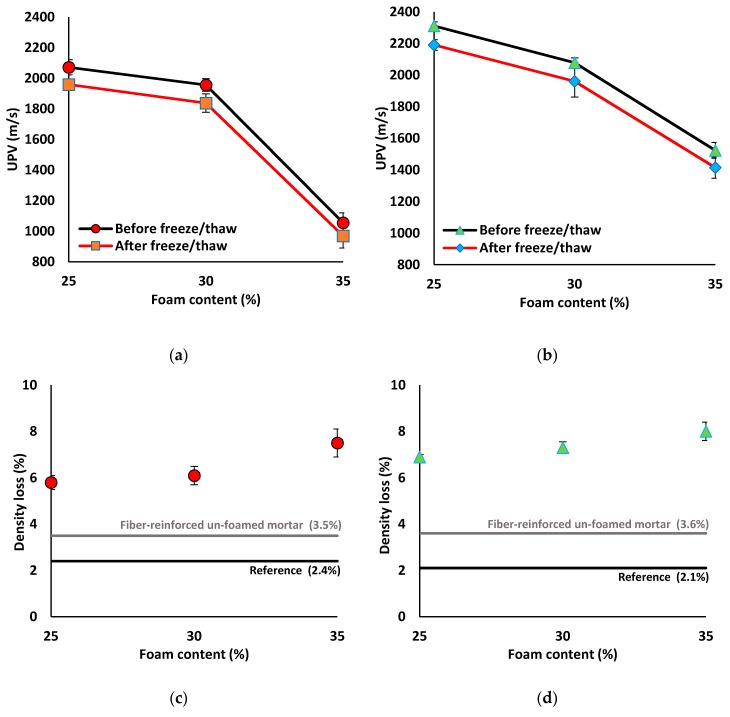
Effects of the freeze/thaw cycles on the UPV results of mix compositions: (**a**) SW50/GW10; (**b**) SW30/GW30; and on density loss (%) of mix compositions: (**c**) SW50/GW10; (**d**) SW30/GW30.

All mix compositions underwent UPV and density reductions after 60 freeze/thaw cycles (see Figure 14 and, Table A2 in Appendix A). The reference mix compositions exhibited ≈2% density and UPV loss after the freeze/thaw test. The addition of PP fibers to the mix compositions resulted in higher UPV and density losses (≈3.5%). Generally, higher UPV and density losses (≈6–8%) were measured in the lightweight mix compositions compared to the normal weight mix compositions. The UPV and density losses detected in the lightweight mortar were increased by the foam content. The density loss due to the freeze/thaw cycles is mainly related to the evaporation of the pore water, and therefore specimens with higher water content (due to higher foam content) showed a higher rate of evaporation. Therefore, the maximum UPV and density loss of around 8% was found in the specimens containing 35% foam content, regardless of the SW/GW ratio. Lightweight mortar with 25% of foam content had 6% of UPV and density loss after 60 freeze/thaw cycles.

**Figure 15:**
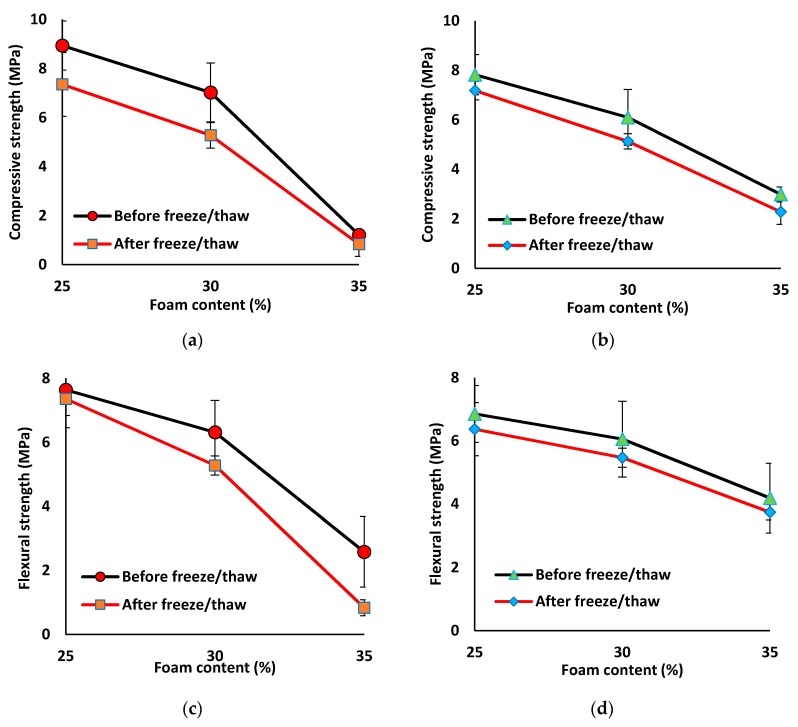
Influences of the freeze/thaw cycles on the compressive strength of mix compositions: (**a**) SW50/GW10; (**b**) SW30/GW30; and on the flexural strength of mix compositions: (**c**) SW50/GW10; (**d**) SW30/GW30.

The compressive and flexural strength of all mix compositions reduced after experiencing 60 freeze/thaw cycles (see Figure 15). The strength losses were in the line with UPV and density losses. The reference and fiber-reinforced normal-weight mix compositions showed the minimum reduction of around 6% in the compressive and flexural strengths due to 60 freeze/thaw cycles. However, the lightweight mortar revealed higher compressive and flexural strength reductions of ≈8–30% and ≈7–17%, respectively. Increasing the foam content in the lightweight mortar resulted in lowering the freeze/thaw resistance and therefore leading to higher compressive and flexural losses. The maximum compressive strength reductions of around 30% (0.8 MPa) and 24% (2.3 MPa) were found in the mix compositions of 35% foam content and SW/GW ratios of 5 and 1, respectively. Similarly, the maximum flexural strength losses of 17% (2.1 MPa) and 10% (3.7 MPa) were detected at the same aforementioned 35% foam content mix compositions, respectively.

The lower freeze/thaw resistance of the lightweight mortar is due to its higher humidity absorption because of a higher permeable porosity, and therefore higher damage to the internal structure due to the increased volume of frozen water. It was proved in [71] that internal structure damage is mainly because of the increased volume of frozen water. Decreasing the freeze/thaw resistance by increasing the foam content can demonstrate the inverse relationship between water absorption and freeze/thaw resistance, since higher water absorption was noticed at higher foam content levels. Additionally, by raising the foam content, the open porosity was increased (see Section 3.2.5), and it was reported in [33] that specimens with closed pore structure showed a higher freeze/thaw resistance. Moreover, with a higher foam content, the pore wall becomes thinner and its load capacity to the stress generated by the frozen water will be lower. Therefore, the higher foam content mix compositions have lower resistance against the development of micro-cracks under the extra stress generated due to the increased volume of frozen water. In addition, cracking the pore walls will also increase pore interconnectivity, which adversely affected the strength and permeable porosity of the lightweight mortar. The negative impacts of pore interconnectivity on the strength of the lightweight concrete were also reported in [21,72,73].

#### 3.3.3. High-Temperature Resistance

**Figure 16:**
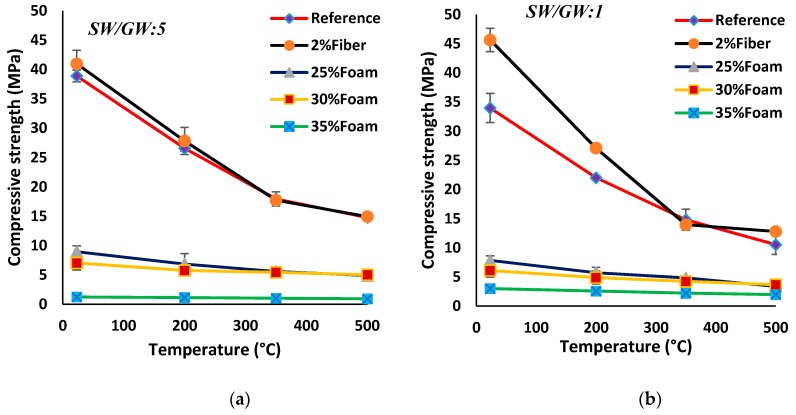

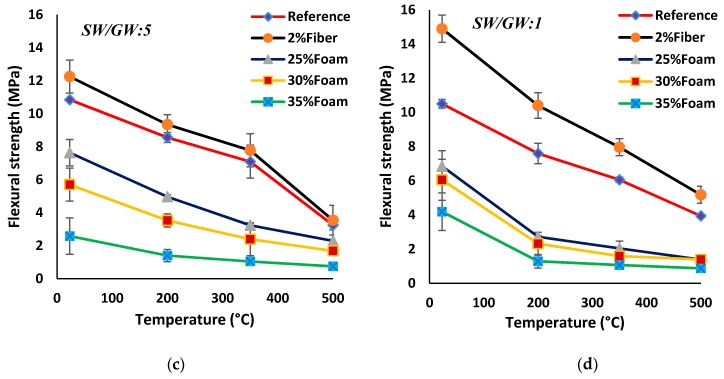
Impact of high temperatures (200, 350, 500 °C) on the compressive strength of mix compositions: (**a**) SW50/GW10; (**b**) SW30/GW30; and on the flexural strength of mix compositions: (**c**) SW50/GW10; (**d**) SW30/GW30.

Reductions in the compressive and flexural strengths due to the high temperatures (200, 350, and 500 °C) were shown in Figure 16. All mix compositions registered a reduction in the mechanical properties after exposing them to high temperatures. An increase in the compressive and flexural strength reductions was observed with an increase in the temperatures. According to the results, SW50/GW10-Reference showed 30%, 54%, and 60% reductions in compressive strength after heating to 200, 350, and 500 °C, respectively. Additionally, around 20%, 34%, and 70% reductions in the flexural strength were recorded in SW50/GW10-Reference after exposure to 200, 350, and 500 °C, respectively. Losses of the strength of about 5–10% were detected by decreasing the SW content in the SW30/GW30-Reference mix composition. The inclusion of the PP fibers in the normal-weight mix compositions increased the compressive and flexural reductions due to the high temperatures. The maximum compressive strength reductions of around 64% (15 MPa) and 72% (13 MPa) were registered at SW50/GW10-2% Fiber and SW30/GW30-2% Fiber, respectively, after exposure to 500 °C, as compared to their unheated state. The maximum loss in flexural strength of around 70–80% was detected at all normal weight and lightweight mix compositions after exposing to the 500 °C.

The lightweight mix compositions resulted in lower compressive strength reductions (8–55%) than the normal-weight mortar (30–72%) after exposure to the test temperatures. Regarding the lightweight mix compositions, the compressive strength reductions were controlled by the density and foam content. The lower-density and higher-foam content mix compositions resulted in a higher thermal resistance and therefore lower loss in compressive strength. Comparable findings were also reported in [13,14,74,75]. The minimum compressive strength reductions of around 8% (1.11 MPa), 18% (0.99 MPa), and 25% (0.90 MPa) were measured at the lowest-density (initial density: 770 kg/m^3^) mix composition (SW50/GW10, 2% Fiber, 35% Foam) after exposure to 200, 350, and 500 °C, respectively, as compared to the unheated state. The SW50/GW10-2% Fiber-25% Foam mix composition (initial density: 1460 kg/m^3^) revealed a rate of thermal resistance in terms of compressive strength reduction two times lower than SW50/GW10-2% Fiber-35% Foam. SW30/GW30-2% Fiber-35% Foam (initial density: 1060 kg/m^3^) showed thermal resistance of 1.5 times higher than SW30/GW30-2% Fiber-25% Foam mixture (initial density: 1510 kg/m^3^). The lower compressive strength reductions of the lightweight mortar can be attributed to the internal permeable porosity paths in the specimens up to the surface. These paths facilitate the releasing of the water vapor to the surroundings and therefore decrease the internal pressure and damage to the matrix.

On the other hand, a different trend was observed with the flexural strength, where the lightweight mix compositions exhibited a higher flexural strength reduction (35–70%) than normal weight mix compositions (20–45%) after heating to 200 and 350 °C. Nevertheless, no differences in flexural strength loss (70–80%) between the normal weight and lightweight mix compositions were observed after heating to 500 °C. The higher flexural strength reduction of lightweight mortar is mainly due to the loss of the PP fibers during the heating process. The high initial flexural strength of the unheated specimens was attributed to the bridging action of PP fiber, and therefore, with melting PP fibers under high temperatures; the advantages of PP fibers in transferring the tensile stress across the crack were eliminated, and thus extensive reductions in the flexural strength were noticed.

### 3.4. Assessment of Porous Structure

**Figure 17:**
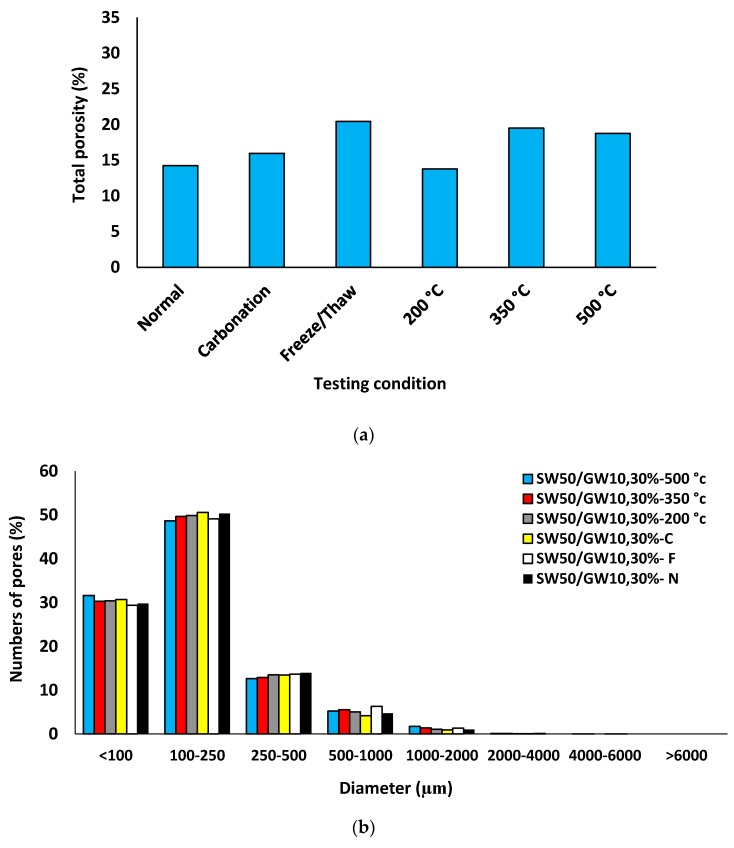

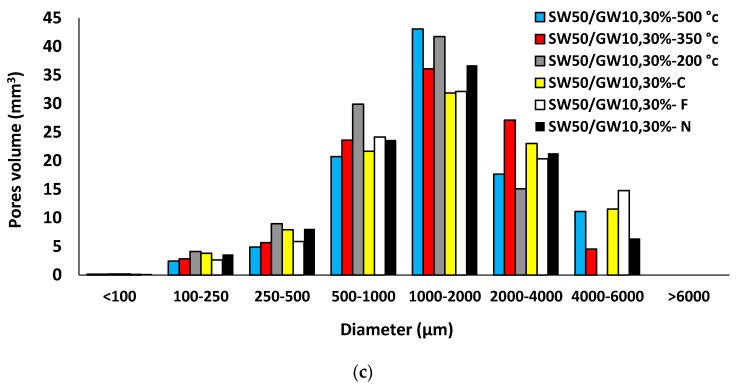
Porous structure characteristics of SW50/GW10-2% Fiber-30% Foam after exposure to different testing conditions: (**a**) total porosity; (**b**) numbers of pores; (**c**) pore volume.

**Figure 18:**
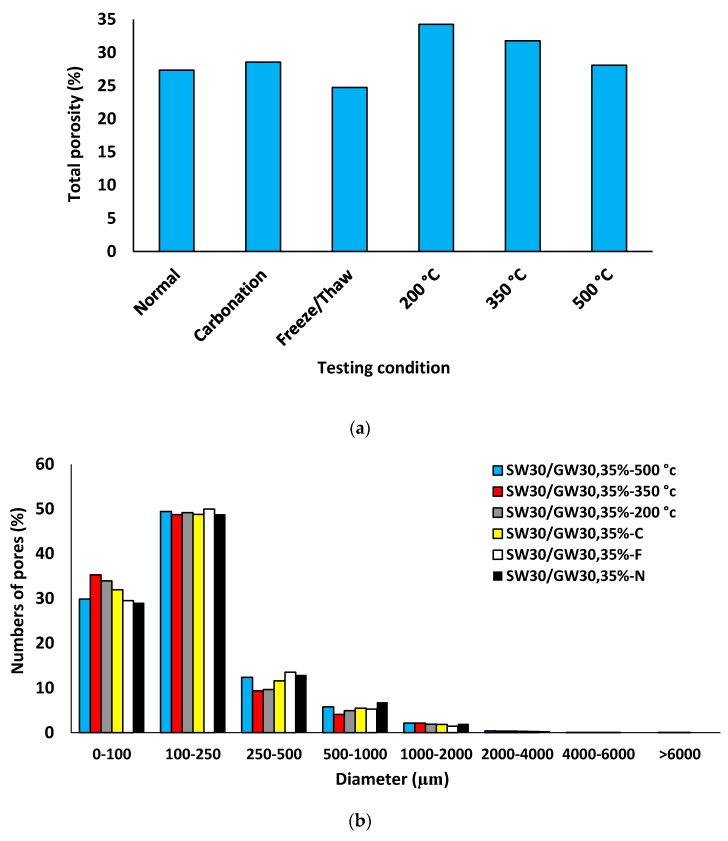

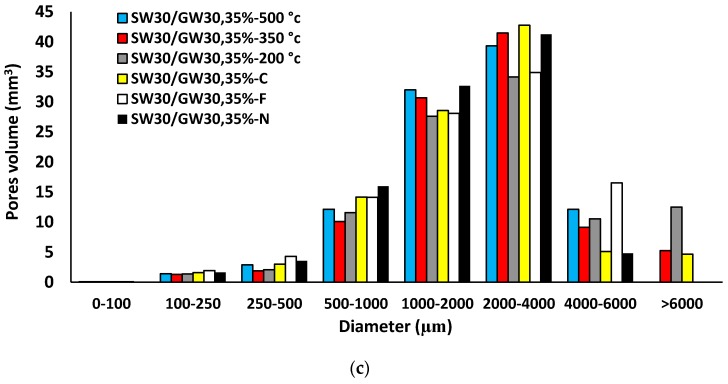
Porous structure characteristics of SW30/GW30-2% Fiber-35% Foam after exposure to different testing conditions: (**a**) total porosity; (**b**) numbers of pores; (**c**) pore volume.

**Figure 19:**
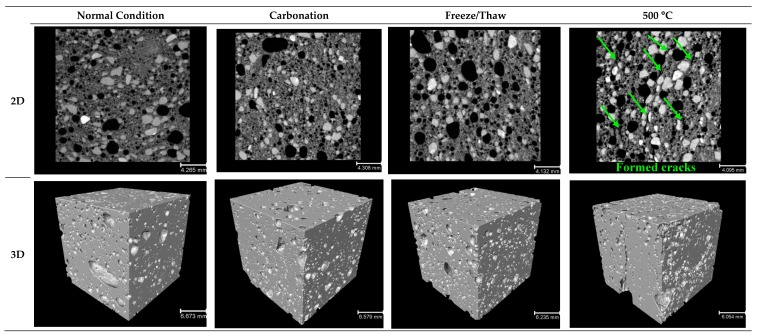
Representative 2D and 3D reconstructions of SW50/GW10-2% Fiber-30% Foam, showing the impact of different test conditions on the porous structure.

**Figure 20:**
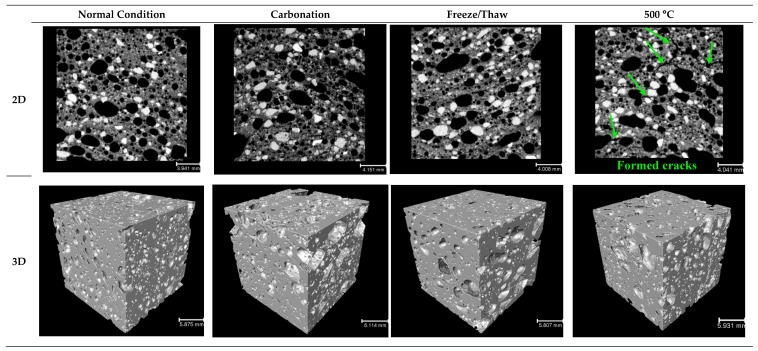
Representative 2D and 3D reconstructions of SW30/GW30-2% Fiber-35% Foam, showing the effects of different test conditions on the porous structure.

Figure 17, Figure 18, Figure 19 and Figure 20 depict the porous characteristics of two selected mixtures (SW50/GW10-2% Fiber-30% Foam and SW30/GW30-2% Fiber-35% Foam) under ambient (or normal) and various harsh conditions. These two mixtures reported both low density and high strength. Therefore, these two mix compositions were chosen for analysis by CT scan technique. Regarding the results presented in Figure 17a and Figure 18a, total porosity was governed by the SW/GW ratio and foam content. The maximum porosities within the mixtures with SW/GW:5 and SW/GW:1 were recorded at more than 20% and 35% when exposed to freeze/thaw cycles and high temperatures (200 °C), respectively. Moreover, it indicates that the performance of the mix compositions containing different SW/GW ratios and foam contents may not behave similarly under different harsh conditions. For instance, in specimen SW50/GW10-2% Fiber-30% Foam, elevating the temperature up to 200 °C led to shrinking due to evaporation of free water in the mixtures. The large volumetric changes, due to temperature, can result in major damage to internal porosity and increase total porosity, as recorded in SW30/GW30-2% Fiber-35% Foam. Smaller volumetric changes reduce the total porosity compared to the mixture exposed to the ambient condition, as measured for SW50/GW10-2% Fiber-30% Foam. Above this temperature, the internal pores were connected due to the imposed damages (see observed cracks in Figure 19 and Figure 20) and the total porosity increased. Different pore characteristics were observed in SW30/GW30-2% Fiber-35% Foam exposed to elevated temperatures.

Interestingly, it was also observed that using different SW/GW and foam contents had no great impact on the numbers of pores, as indicated in Figure 17b and Figure 18b. In both mix compositions, regardless of the harsh condition assessments, 30% of the pores had a diameter of ≤100 μm, more than 50% of pores had a diameter between 100–250 μm, 10% of the pores had a diameter of 250–500 μm, and the rest had a diameter 500–2000 μm.

Moreover, it was noticed that different SW/GW ratios and foam content have a strong effect on three-dimensional pore volumes. According to the results presented in Figure 17c and Figure 18c, 30–40% of the largest pores (by volume) in specimen SW50/GW10-2% Fiber-30% Foam varied in the diameter ranges of 1000–2000 μm, and 20–30% of the largest pores changed in the diameter range of 500–1000 μm. 40–50% of the largest pores in specimen SW30/GW30-2% Fiber-35% Foam were in the range of 2000–4000 μm in diameter, and 25–35% of the largest pores are in the range of 1000–2000 μm in diameter. The impact of exposing the developed lightweight wool-based alkali activated mortars to harsh conditions was illustrated as three and two-dimensional views in Figure 19 and Figure 20. Obviously, comparing the results indicates that SW30/GW30-2% Fiber-35% Foam has higher porosity than SW50/GW10-2% Fiber-30% Foam. Moreover, it was observed that some cracks formed and propagated in the mix compositions caused by exposure to high temperatures (500 °C), which explains the loss of strength at this temperature.

## 4. Conclusions

This study presents the results of experiments on the production of fiber-reinforced lightweight mineral wool-based alkali activated mortars. Fresh and hardened-state properties of the developed lightweight alkali activated mortars were investigated.

Increasing the stone-to-glass-wool ratio in the fiber-reinforced alkali activated mortar results in higher workability. Moreover, an inverse relationship is detected between workability and hardened density of the developed mix compositions. A linear correlation is observed between strength and density. The increasing rate of strength due to an increase of density is higher in SW50/GW10, as compared to SW30/GW30.

The developed fiber-reinforced lightweight SW/GW-based alkali activated mortar with 25, 30, and 35% foam content indicates that the compressive and flexural strengths are in a range of 1–9 MPa and 2.6–8 MPa, respectively, and that strength decreases with an increase of foam content.

Interestingly, the developed lightweight mortars have a lower drying shrinkage rate than mix compositions without foam, regardless of SW/GW ratio, so that the lowest drying shrinkage rate of ≈1.7% is measured in the mix compositions with 35% foam content.

Moreover, it was found that increasing SW content has a greater impact on increasing the efflorescence for all mix compositions. The efflorescence increases with increasing foam content, regardless of SW and GW contents.

Water absorption, permeable porosity, and high-temperature resistance are inversely proportional to the density, while freeze-and-thaw resistance is directly proportional to the density.

After experiencing carbonation, it was detected that density and strength increase for the developed lightweight alkali activated mortars, as compared to the mix composition without foam. The increases are directly proportional to the level of foam content. Additionally, total porosity and pore volumes are governed by the SW/GW ratios and foam contents, while these parameters have low impacts on the number of pores.

Promisingly, using different SW/GW ratios had no great impacts on the mechanical and durability properties of the lightweight materials. Therefore, this study promotes and encourages the use of mineral wools in the production of lightweight panels for indoor applications due to its environmental friendly impacts and cost-effectiveness.

## Figures and Tables

**Table 1:** The chemical compositions and densities of SW, GW, and MK.

Material	Element/Oxides (%, *w*/*w*)											Density (g/cm^3^)
	CaO	SiO_2_	Al_2_O_3_	Fe_2_O_3_	Na_2_O	K_2_O	MgO	P_2_O_5_	TiO_2_	SO_3_	Cl	
**SW**	17.40	40.40	15.80	9.20	1.40	0.40	12.60	0.10	0.80	-	-	2.90
**GW**	7.10	62.40	1.80	0.60	16.80	0.90	2.20	-	-	0.90	0.10	2.50
**MK**	0.10	51.78	38.27	1.86	0.17	2.27	1.13	0.11	0.09	0.10	-	2.60

**Table 2:** The physical and mechanical properties of the PP fiber [40].

Fiber Type	Length/ Diameter (mm/mm)	Elastic Modulus (GPa)	Tensile Strength (MPa)	Elongation at Break (%)	Density (g/cm^3^)
**PP**	833	9.6	910	<12	0.91

**Table 3:** Mix compositions design (%mass).

Mixture	$\frac{\mathrm{SW}}{B^{\left[a\right]}}$	$\frac{\mathrm{GW}}{B}$	$\frac{\mathrm{MK}}{B}$	$\frac{A^{\left[b\right]}}{B}$	$\frac{{\mathrm{SS}}^{\left[c\right]}}{{\mathrm{SH}}^{\left[d\right]}}$	NaOH Concentration	$\frac{\mathrm{Sand}}{B}$	PP Fiber Content	Foam Content
SW50/GW10-Reference	50	10	40	0.88	2.5	10 M	1	0	0
SW50/GW10-2% Fiber								2	0
SW50/GW10-2% Fiber-25% Foam									25
SW50/GW10-2% Fiber-30% Foam									30
SW50/GW10-2% Fiber-35% Foam									35
SW30/GW30-Reference	30	30	40					0	0
SW30/GW30-2% Fiber								2	0
SW30/GW30-2% Fiber-25% Foam									25
SW30/GW30-2% Fiber-30% Foam									30
SW30/GW30-2% Fiber-35% Foam									35

B^[a]^: Binder, A^[b]^: Alkali activator, SS^[c]^: Sodium silicate, SH^[d]^: Sodium hydroxide.

## Appendix A

**Table A1:** Effects of carbonation on the hardened state properties of the reference and fiber reinforced mixtures (no foam).

Mixture	Density (kg/m^3^)		UPV (m/s)		Compressive Strength (MPa)		Flexural Strength (MPa)	
	Before	After	Before	After	Before	After	Before	After
**SW50/GW10-Reference**	1860	1867	2916	2998	39.0	41.0	10.8	12.0
**SW50/GW10-2% Fiber**	1794	1803	2793	2890	43.5	46.6	12.3	13.7
**SW30/GW30-Reference**	1880	1887	2857	2920	34.0	37.0	10.5	11.6
**SW30/GW30-2% Fiber**	1824	1832	2783	2870	45.6	50.3	15.0	16.6

**Table A2:** Effects of Freeze/thaw tests on the hardened state properties of the reference and fiber reinforced mixtures (no foam).

Mixture	UPV (m/s)		Compressive Strength (MPa)		Flexural Strength (MPa)	
	Before	After	Before	After	Before	After
**SW50/GW10-Reference**	2870	2814	39.0	36.7	10.8	10.1
**SW50/GW10-2% Fiber**	2776	2703	40.6	43.5	12.3	11.4
**SW30/GW30-Reference**	3100	3036	34.0	32.0	10.5	10.0
**SW30/GW30-2% Fiber**	2980	2896	45.6	42.7	15.0	14.1
